# A weary soul needs to recover! A study on the impact of supervisor support for recovery on employees’ thriving at work and job satisfaction

**DOI:** 10.3389/fpsyg.2025.1725037

**Published:** 2026-01-12

**Authors:** Zhaoyang Liu, Changchun Gao, Chenhui Yu

**Affiliations:** Glorious Sun School of Business and Management, Donghua University, Shanghai, China

**Keywords:** intrinsic need, job burnout, job demand, job satisfaction, supervisor support for recovery, thriving at work

## Abstract

While existing research has provided preliminary insights into the role of SSR in workplace wellbeing, these studies have not systematically differentiated the effects of SSR across distinct dimensions of workplace wellbeing, nor have they fully elucidated the underlying mechanisms or contextual boundary conditions. To address these gaps, this study conducted a questionnaire survey of 429 employees from 10 organizations and analyzed the data using latent moderated structural equations (LMS), enabling the examination of complex moderating effects within the proposed model. We found the following conclusions: First, SSR has a positive direct impact on employees’ thriving at work but it does not have a significantly direct effect on job satisfaction. Second, job burnout and intrinsic needs, respectively, mediate the relationships between SSR and job satisfaction and the relationship between SSR and thriving at work. Third, job demand positively moderates the relationship between SSR and job burnout but it does not moderate the relationship between SSR and intrinsic need. This work enriches the theoretical research on SSR in positive organizational behavior and also provides practical implications for the team leadership style and time management of employees in enterprises.

## 1 Introduction

The data from Mercer Marsh Benefits’ (MMB) “*Employee Benefits 2023 Workplace Wellbeing Report*” indicates that nearly half (47%) of employees feel stressed in their daily lives, and over half (52%) have felt mentally unwell while working in the past year (Mercer Marsh Benefits, 2023). Meanwhile, a significant proportion of employee reported experiencing high levels of fatigue despite engaging in relatively low levels of physical labor at work (Mercer Marsh Benefits, 2023). Unfortunately, only 37% of employers say they will design work with employees’ wellbeing in mind, such as ensuring reasonable workloads, reducing complexity, and implementing no-meeting days. Excessive work pressure and a lack of job autonomy may harm employees’ mental health, which is an important component of their wellbeing in the workplace. The wellbeing of workers is not only a core social goal that the vast majority of governments focus on, but also a key indicator for measuring organizational success and leadership effectiveness (Serafim et al., 2024). For promoting the continuous prosperity of organizations, it is significant to explore the intrinsic connection between leaders’ support for employee recovery and employee wellbeing is of great significance.

In positive psychology, subject wellbeing is divided into hedonic wellbeing and eudaimonic wellbeing. Thriving at work belongs to eudaimonic happiness, emphasizing spiritual growth and the pursuit of value (Wang et al., 2023; Karadas et al., 2025). Thriving at work is a psychological state characterized by the simultaneous experience of vitality and ongoing learning in the workplace. It serves as a key indicator of individual growth and developmental progress (Kleine et al., 2019). Individuals exhibiting this positive state demonstrate high levels of energy, are willing to invest greater effort in their work, and engage in continuous learning and self-improvement. Job satisfaction is a key component of hedonic wellbeing, representing an individual’s affective responses and emotional experiences toward their work (Ziegler and Schlett, 2013; Lee and Bae, 2024). It reflects a cognitive evaluation of various job-related attributes, such as job characteristics, compensation, opportunities for advancement, relationships with supervisors, and interactions with colleagues. The relevant literature of positive organizational behavior has discussed in detail the influencing factors of job satisfaction and thriving at work from the perspectives of salary level, organizational culture, human resource management, and leadership. At the individual level, income, personality, and motivation serve as key determinants of employees’ job satisfaction and thriving at work (Jiang et al., 2019; Ni et al., 2023; Lin et al., 2020; Paterson et al., 2014; Han et al., 2024). At the team level, factors such as role identity, leader-member exchange, and knowledge sharing significantly influence job satisfaction and thriving at work. At the organizational level, cultural inclusiveness, organizational diversity, and human resource practices—including training programs and incentive systems—contribute to enhanced levels of employee job satisfaction and thriving at work (Alwahhabi et al., 2023; Ghazzawi et al., 2025).

Supervisor support for recovery (SSR) may have a significant impact on employees’ wellbeing in the workplace. Recovering from work stress—defined as the relaxation process of reducing or eliminating stress caused by work-related stressors (Meijman and Mulder, 1998)—is crucial for helping employees maintain wellbeing, health and performance (Steed et al., 2021). SSR refers to the extent to which supervisors support employees’ recovery experiences. It requires supervisors to limit work pressure or expectations during non-working hours and proactively provide recovery support (Zhu et al., 2025). In the subsequent concept refinement and scale development, SSR consists of four best practices: First, avoiding communicating about work during non-working hours. Second, avoiding requiring work during non-working hours. Third, modeling recovery. Fourth, encouraging recovery. These studies have initially verified the positive role of SSR in alleviating work pressure and improving the physical and mental health of employees (John and Ryan, 2025; Sabine et al., 2024).

A substantial body of literature has examined the antecedents of job satisfaction and thriving at work, providing comprehensive insights into strategies for enhancing employees’ wellbeing in the workplace (Bernini and Tampieri, 2025; Chen et al., 2023; Okros and Virga, 2025; Wei et al., 2025). Furthermore, scholars have clarified the conceptual foundations of supervisor support for recovery, developed validated measurement scales, and begun to investigate its psychological effects (Zhu et al., 2025; John and Ryan, 2025; Sabine et al., 2024). Nevertheless, two key limitations remain. First, although numerous studies have separately explored the mechanisms underlying job satisfaction and thriving at work, few have adopted an integrative perspective—simultaneously drawing on hedonic and eudaimonic frameworks—to identify their shared determinants (Petts et al., 2025; Qamar et al., 2024). Second, research on the psychological impact of SSR is still in its early stages, with limited empirical evidence supporting its beneficial role (Zhu et al., 2025; John and Ryan, 2025; Sabine et al., 2024). Therefore, further validation of the relationship between SSR and employee wellbeing is warranted.

In recent years, a large number of positive psychology literature has attempted to study the causes of individual wellbeing from a dual perspective of negative and positive. Therefore, following this trend, we established a dual-path model of how SSR affects employees’ job satisfaction and thriving at work based on the JD-R model and self-determination theory. For this purpose, we conducted a questionnaire survey of 429 employees from 50 departments (or teams) in 12 companies and verified the research hypotheses using latent moderated structural equation modeling (LMS). The research conclusions of this paper enrich the theoretical research of positive organizational behavior on employees’ wellbeing in the workplace and contribute management guidance for the best practices of SSR in manufacturing enterprises. These research efforts can be summarized into the following core questions:

*Q1*: Will SSR enhance employees' job satisfaction and thriving at work?

*Q2*: If the effect exists, how does SSR influence job satisfaction and thriving at work?

*Q3*: How does the mechanism of action of SSR change under different intensities of work requirements?

## 2 Theory and hypothesis

### 2.1 Theoretical analysis

#### 2.1.1 JD-R model

According to the Job Demands-Resources (JD-R) model, job characteristics can be categorized into two broad dimensions: job demands and job resources (Bakker and Demerouti, 2024). Job demands refer to those aspects of a job that require sustained physical, psychological, or social effort and are associated with physiological and psychological costs, such as work overload, role conflict, time pressure, and job insecurity (Choubey and Agrawal, 2025). Job resources, on the other hand, encompass physical, psychological, social, or organizational aspects of the job that facilitate the achievement of work goals, reduce job demands, and promote personal growth, learning, and development (Demerouti et al., 2001). Building upon its core framework, the JD-R model proposes a dual-process mechanism—namely, the health impairment process and the motivational process. The health impairment process is triggered by high job demands and insufficient job resources, leading to strain and burnout, which in turn result in adverse organizational outcomes (Bakker et al., 2023). Conversely, the motivational process is activated by abundant job resources, fostering increased work engagement and yielding positive individual and organizational consequences (Bakker and Demerouti, 2017).

#### 2.1.2 Self-determination theory

Self-Determination Theory (SDT), proposed by Richard M. Ryan and Edward L. Deci in the early 1970s, is a highly significant theory in the field of psychology. As a novel theory of motivation, SDT emphasizes the degree of self-determination in human behavior, viewing motivation as a continuum based on the level of self-determination. It posits that the social environment can enhance human motivation by supporting the satisfaction of three intrinsic needs: autonomy, competence, and relatedness (Deci et al., 2017). The theory posits that extrinsic demands often lead individuals to experience a sense of control and diminished psychological ownership, whereas the fulfillment of intrinsic needs—such as autonomy, competence, and relatedness—is more strongly associated with enhanced wellbeing and internalized motivation.

### 2.2 Hypothetical development

#### 2.2.1 The impact of SSR on employees’ thriving at work and job satisfaction

Job satisfaction refers to the degree to which employees are content with aspects such as job content, working environment, salary and benefits, and career development (Cammann et al., 1983; Judge et al., 1998). Thriving at work refers to a psychological state in which employees experience the integration of vitality and learning during work, characterized by heightened work engagement and perceived personal growth, and has been shown to significantly enhance innovation performance (Porath et al., 2012). Humanistic theory holds that organizations can enhance employees’ job satisfaction and thriving at work by fulfilling their needs for social interaction, self-esteem, and self-actualization (Criswell, 2003; DeRobertis, 2015). Empirical research indicates that a relaxed cultural atmosphere and enriched job design (increasing authority and reengineering processes) can achieve these goals and improve employees’ subjective wellbeing (To et al., 2020; Martela, 2025).

As an optimal organizational practice, SSR is defined as the tendency of supervisors to avoid behaviors that hinder recovery and engage in behaviors that are beneficial to employees’ recovery. According to the Effort Recovery Model (ERM), recovery occurs when the short-term load response decreases due to the cessation of energy consumption, and individuals’ life satisfaction, vitality, resilience, self-efficacy and hope will all increase accordingly (Zhu et al., 2025). To examine the predictive validity of SSR, Zhu et al. (2025) employed a mixed-methods research design and found that SSR fosters multiple positive outcomes, including leaders’ health-oriented behaviors, high-quality leader-member exchange, and employees’ restorative experiences, thereby supporting its positive association with employees’ job satisfaction (Zhu et al., 2025). John and Ryan (2025) found in the pilot evaluation of the restorative leadership training program that leaders’ commitment to restoration can enhance employees’ authenticity and sense of efficacy, thereby stimulating their instinct to pursue their ideal selves (John and Ryan, 2025). This evidence supports the promoting effect of SSR on employees’ thriving at work.

*H1*: SSR has a positive direct impact on employees' job satisfaction.

*H2*: SSR has a positive direct impact on employees' thriving at work.

#### 2.2.2 The mediating effect of job burnout and intrinsic needs

Job burnout refers to the state of physical and mental fatigue and exhaustion experienced by an individual under heavy work pressure, consisting of three elements: emotional exhaustion, depersonalization and low sense of accomplishment (Maslach, 1986; Maslach and Jackson, 1981). Demerouti et al. (2001) proposed the JD-R model in 2001 and used it to explain job burnout. According to the JD-R model, job burnout has long been associated with work pressure. An increase in job resources or a reduction in job demands may alleviate employees’ job burnout. Schaufeli and Taris (2014) conducted a review of eight longitudinal studies involving workers across different countries and found that five provided supports for the JD-R model’s predictions regarding the causal relationships between job demands, job resources, employee wellbeing, and job burnout.

The concept of SSR emphasizes two aspects—reducing work demands during non-working hours and increasing the support from supervisors for recovery. According to the JD-R model, the first connotation of SSR limits the work tasks of employees during non-working hours, reducing the loss of their psychological energy. The second connotation of SSR improves employees’ psychological capital, human capital and social capital, which increase the resources needed for them to cope with work and life. In the fourth to sixth studies of Zhu et al. (2025), empirical results supported the negative impact of SSR on employees’ psychological detachment and the positive impact of SSR on work-family balance and recovery experiences. Additionally, Sabine et al. (2024) found through diary data from 152 employees that SSR has a negative impact on psychological detachment and emotional exhaustion, and promotes empathy for recovery and respect for boundaries (Deci and Ryan, 1985; Ryan and Deci, 2000).

*H3*: SSR has a negative impact on job burnout.

Job burnout is composed of three elements: emotional exhaustion, depersonalization, and low sense of accomplishment. According to the hedonic wellbeing definition formula, emotional exhaustion and depersonalization can reduce happiness by suppressing positive emotions and triggering negative emotions. According to Bandura’s self-efficacy theory, depersonalization and low sense of accomplishment are usually associated with low self-efficacy, and self-efficacy is an important prerequisite for individual vitality and learning. Maslach (1986) and Maslach and Jackson (1981) supported the negative effect of job burnout on learning motivation, self-efficacy, and effectiveness, as well as the positive effect of job burnout on work distress, work pressure, and negative attitudes.

*H4*: Job burnout has a negative impact on employees' job satisfaction.

*H5*: Job burnout has a negative impact on employees' thriving at work.

Integrating hypotheses H3 to H5, we establish the theoretical framework of “SSR—job burnout—job satisfaction and thriving at work.” Grounded in the dual-path mechanism of the Job Demands-Resources (JD-R) model, work intensity can exacerbate employees’ job burnout, while work resources can alleviate it (De Beer et al., 2024; van Zyl et al., 2024). Meanwhile, SSR contributes to building employees’ psychological capital and mitigating work-related demands during non-work time, thereby alleviating job burnout and subsequently enhancing subjective wellbeing (Zhu et al., 2025). Thus, SSR has a positive influence on job burnout. Job satisfaction represents a comprehensive assessment by employees of various job-related attributes. Empirical studies conducted by Song et al. (2020) and Galanis et al. (2025) have demonstrated that job burnout, as a cumulative outcome of adverse job characteristics, is negatively associated with job satisfaction. Under the JD-R model, De Beer et al. (2024) found a negative correlation between job burnout and growth factors during the process of validating the scale’s predictive validity.

*H6*: Job burnout plays a mediating role between SSR and employees' job satisfaction.

*H7*: Job burnout plays a mediating role between SSR and employees' thriving at work.

According to self-determination theory, intrinsic needs consist of three fundamental components: competence, relatedness, and autonomy, which serve as key drivers of individual behavior. It is widely accepted among scholars that the influence of management practices on job satisfaction and thriving at work is mediated through the fulfillment of these intrinsic needs (Demerouti et al., 2001). Consequently, a management practice can only be deemed effective when the necessary motivational conditions are met, thereby enhancing employee wellbeing (Paterson et al., 2014).

SSR consists of four elements: avoiding work-related demands during non-working hours, refraining from work communication during non-working hours, modeling recovery, and encouraging recovery. In a busy state without recovery, employees’ work motivation is more determined by external conditions such as task pressure, leaders’ urging, customer demands, and salary benefits. Employees lack autonomy and often feel controlled. The purpose of SSR is to enhance employees’ recovery experience. During the practice of SSR, employees truly feel that time belongs to them and can communicate with colleagues on many non-work topics to enhance their friendship. The statistical results of Zhu et al. (2025) and John and Ryan (2025) support the positive impact of SSR on LMX, employee relationship quality, self-efficacy, and psychological ownership.

*H8*: SSR has a positive impact on employees' intrinsic needs.

According to self-determination theory, intrinsic needs play a decisive role in an individual’s evaluation and behavioral motivation (Bakker and Demerouti, 2017). From the perspective of hedonistic wellbeing, intrinsic needs reflect a higher level of personal pursuit for employees compared to extrinsic needs. The satisfaction of intrinsic needs can trigger pleasant emotional experiences for employees and promote their positive evaluation of work (Paterson et al., 2014). From the perspective of eudaimonic wellbeing, intrinsic needs are associated with goals such as self-actualization, social interaction, and self-esteem. The satisfaction of intrinsic needs can motivate employees to study hard, improve their physical and mental health, and enhance their work vitality (Kleine et al., 2019; Paterson et al., 2014).

*H9*: Intrinsic needs have a positive impact on employees' job satisfaction.

*H10*: Intrinsic needs have a positive impact on employees' thriving at work.

Integrating hypotheses H8 to H10, we establish the theoretical pathway of “SSR → Intrinsic Needs → Job Satisfaction and thriving at work.” Grounded in Self-Determination Theory, SSR enhances employee vitality and wellbeing by satisfying fundamental psychological needs. This mediating role of intrinsic needs—specifically relatedness, competence, and autonomy—is supported by empirical evidence from Zhu et al. (2025) and John and Ryan (2025), who confirm their function in linking SSR to improved employees’ intrinsic need. Li et al. (2024) found that intrinsic motivation has a positive impact on thriving at work, organizational commitment, and job satisfaction to help nurses get out of the predicament of high workload and unpleasant work experience. These pieces of evidence indicate that SSR exerts a positive influence on job satisfaction and thriving at work through intrinsic motivation.

*H11*: Intrinsic needs play a mediating role between SSR and job satisfaction.

*H12*: Intrinsic needs play a mediating role between SSR and thriving at work.

#### 2.2.3 The moderating effect of task complexity

**Figure 1:**
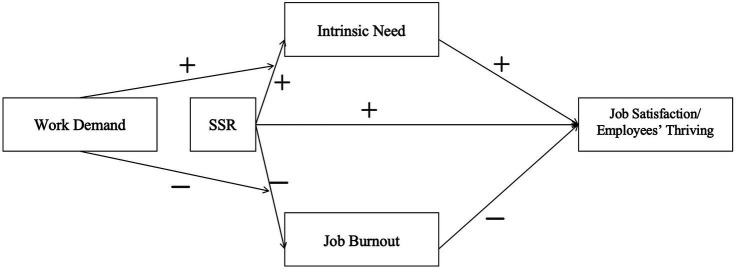
Theoretical model.

Sabine et al. (2024) found that the effectiveness of SSR is constrained by the external environment. Only when work design factors or leadership factors support SSR can the authenticity of SSR practices be ensured, thereby fully realizing the role of SSR. According to the JD-R model, job burnout is jointly influenced by job demands and job resources. While SSR enhances employees’ psychological and social capital, excessively high job demands may exacerbate work-to-nonwork spillover and diminish the effectiveness of SSR in promoting employees’ recovery experiences (Bakker and Demerouti, 2017). Self-determination theory holds that extrinsic motivation and intrinsic motivation are mutually substitutable, and intrinsic motivation is more conducive to promoting individuals’ positive behaviors than extrinsic motivation. However, under high-intensity working conditions, an individual’s work motivation is mainly determined by external motivational factors such as leadership demands, client urging, and salary incentives. Given this, high-intensity working conditions may weaken the effect of SSR on intrinsic needs (Semmer et al., 1995; Figure 1).

*H13*: Job demands positively moderate the relationship between SSR and job burnout.

*H14*: Job demands negatively moderate the relationship between SSR and intrinsic needs.

## 3 Research design

### 3.1 Data

From March 2024 to May 2025, we conducted a survey across 50 departments (or teams) in 10 enterprises. The sample included four manufacturing companies located in Henan and Shandong provinces, four e-commerce companies in Zhejiang and Jiangsu provinces, and two cultural and creative firms in Hunan Province, all within China. Questionnaires were primarily distributed to key functional units within each organization, including finance, production and operations, marketing, human resources (or administration), and technical departments (or R&D). Ten questionnaires were administered to each department or team, resulting in a total distribution of 500 questionnaires. To mitigate common method bias, the questionnaire data were collected in three distinct waves. In the first wave (March–June 2024), employee demographic information and data for the explanatory variable (SSR) were collected. In the second wave (August–December 2024), data on the mediating variables (intrinsic need and job burnout) and the moderating variable (job demand) were gathered. In the third wave (January–May 2025), data pertaining to the dependent variable were obtained. A total of 452 questionnaires were collected. After excluding 23 responses with incomplete answers, completely identical patterns, or failure to select option A on the attention-check item (a filter question designed to ensure respondent attentiveness), 429 valid questionnaires remained for analysis.

### 3.2 Measurement

We employed a five-point Likert scale to measure the key research variables. All measurement items were drawn from well-established and authoritative scales. Following the reliability and validity analyses conducted during the pre-survey, we made appropriate refinements, including minor adjustments and deletions of certain items, to enhance measurement precision and contextual relevance.

(1) Explained variable 1: Thriving at work (TW). We adopted the scale developed by Porath et al. (2012) and Porath et al. (2022) to measure thriving at work from two dimensions: vitality and learning. Among them, vitality is composed of four items: “I feel energetic,” “I look forward to each day,” “I am full of energy,” and “I feel sharp and alert.” Learning is composed of four items: “I am experiencing a rapid development process,” “I am growing actively,” “I am glad to witness the slightest progress of my thoughts,” and “I keep learning as time goes by.”
(2) Explained variable 2: Job satisfaction (JS). We drew upon the established research of Cammann et al. (1983) and Judge et al. (1998) to measure job satisfaction from three key dimensions: salary level, interpersonal relationships, and job characteristics. The scale comprises seven items, namely: “I feel that others respect me,” “I am satisfied with the way others treat me,” “I am satisfied with my interpersonal relationships at work,” “I am satisfied with the benefits I receive,” “I am satisfied with my current salary,” “Most of the time, I am enthusiastic about my job content,” and “I find enjoyment in my work.”
(3) Explanatory variable: Supervisor support for recovery (SSR). We adopted the newly developed scale by Zhu et al. (2025) to measure SSR. The scale comprises four dimensions: refraining from communicating about work during nonwork time, refraining from requiring work during nonwork time, modeling recovery, and encouraging recovery, encompassing a total of 12 items.
(4) Mediating variable 1: Job burnout (JB). We drew upon the established frameworks of Maslach (1986) and Maslach and Jackson (1981) to assess job burnout across three core dimensions: exhaustion, depersonalization, and reduced personal accomplishment. During the pre-survey phase, we observed that the original items measuring reduced personal accomplishment employed reverse scoring, which resulted in suboptimal reliability and validity. To enhance psychometric properties, we revised the scoring approach by adopting positively worded items. The final scale comprises nine items, including “I am extremely tired,” “I do not care about the feelings of my work objects,” “I cannot effectively solve the problems of my work objects,” “I am worried that my work will affect my mood,” “My work objects often complain about me,” “I cannot effectively influence others through my work,” “I often feel exhausted,” “I work with a cynical attitude,” and “I cannot create a relaxed and lively work atmosphere.”
(5) Mediating variable 2: Intrinsic need (IN). We referred to the research of Bakker et al. (2023) and measured intrinsic needs from three aspects: relationship, ability and autonomy, with a total of six items. The scale consists of six items such as “I can make decisions about many aspects of my work,” “I can arrange my working time independently,” “I have a sense of belonging in the team,” “The quality of my relationships is good,” “My knowledge and skills have improved,” and “I have gained rich work experience.”
(6) Moderating variable: Job demands (JD). We employed the scale developed by Deci et al. (2017) to assess job demands. The scale comprises four items: “In my work, I often need to make very complex decisions,” “In my work, I often need to undertake a large number of tasks,” “In my work, there are always new arrangements that challenge my abilities,” and “In my work, the organization imposes strict requirements on our emotional expression.”

### 3.3 Method

Firstly, we employed the Latent Moderated Structural Equations (LMS) model to validate the entire theoretical model by MPlus9.0 (Klein and Moosbrugger, 2000; Kelava et al., 2014). Then, we utilized the Bootstrap method to test the mediating effects of job burnout and intrinsic needs, further ensuring the robustness of the statistical conclusions by SPSS. Finally, we adopted the simple slope plot to test the moderation effect hypotheses and presented the moderating effect of job demands in a visual form by SPSS and EXCEL.

### 3.4 Ethical statement

The ethics committee of the first author’s institution granted an exemption from ethical review for this study. The committee chair provided four justifications: First, the questionnaire content did not include sensitive information. Second, the use of a 5-point Likert scale minimized potential intrusiveness and helped safeguard participant privacy. Third, as a non-experimental survey-based study, the research posed minimal risk to participants’ psychological wellbeing, in contrast to experimental designs that may induce emotional or cognitive stress. Fourth, all participants provided written informed consent prior to their involvement in the study, with signed consent forms retained as supporting documentation.

## 4 Results

### 4.1 Descriptive statistics

Table 1 displays the descriptive statistics results for the main variables across 429 samples. The variance inflation factor (VIF) is reported to assess the extent of multicollinearity among the variables. As all VIF values are below the threshold of 5, multicollinearity does not pose a significant issue in the dataset.

**Table 1:** The results of descriptive statistics.

Variable	N	Max	Min	Mean	SD	VIF
SSR	429	4.75	1.5	3.552	1.027	1.348
IN	429	5	1.167	3.836	0.93	1.304
JB	429	4.667	1	2.07	0.855	3.914
JD	429	5	1	2.047	0.854	3.762
TW	429	5	1.5	3.663	0.989	–
JS	429	5	1.143	3.733	0.875	–

### 4.2 Reliability and validity test

We conducted reliability and validity analyses on all variables using SPSS 27.0. The results showed that Cronbach’s alpha coefficients for all variables exceeded 0.8, the cumulative variance explained by the extracted factors was greater than 60%, AVE values were above 0.5, and composite reliability (CR) values surpassed 0.7, indicating that the measurement scale exhibits satisfactory reliability and construct validity. Furthermore, Harman’s single-factor test was employed to assess potential common method bias. The first unrotated principal component accounted for approximately 32% of the total variance, which is below the commonly accepted threshold of 40%, suggesting that common method bias does not pose a significant threat to the results (Table 2).

**Table 2:** The results of reliability and validity tests.

Variable	Cronbach’s α	Cumulative contribution degree	AVE	CR
SSR	0.945	62.505%	0.6849	0.8968
IN	0.879	62.444%	0.6859	0.8675
JB	0.909	67.976%	0.6929	0.8709
JD	0.804	67.834%	0.7142	0.8821
TW	0.914	62.442%	0.6102	0.8241
JS	0.842	61.448%	0.6264	0.8376

### 4.3 The results of SEM

By running the MPlus9.0, we analyzed the data using the LMS method and obtained the results as shown in Table 3. As shown in Table 3, the *p*-value is 0.08 (*p* > 0.05), the chi-square to degrees of freedom ratio is 1.982 (below the threshold of 3), GFI = 0.949 (above the recommended cutoff of 0.90), RMSEA = 0.048 (less than 0.10), RMR = 0.157 (exceeding the criterion of 0.05), CFI = 0.919 (greater than 0.90), NFI = 0.949 (above 0.90), and NNFI = 0.914 (also exceeding 0.90). With the exception of RMR, all fit indices fall within the acceptable ranges commonly used as reference criteria in structural equation modeling. Therefore, the overall model fit of the LMS analysis can be considered satisfactory.

**Table 3:** The fitting results of the latent moderated structural equation model.

Index	χ^2^	df	*P*	χ^2^/df	GFI	RMSEA	RMR	CFI	NFI	NNFI
Reference standards	–	–	>0.05	<3	>0.9	<0.10	<0.05	>0.9	>0.9	>0.9
Actual value	1938.48	978	0.08	1.982	0.949	0.048	0.157	0.919	0.949	0.914

Table 4 presents the estimated results of the path coefficients among the variables in the LMS model. The standardized path coefficient of SSR → TW is 0.322 (*p* < 0.000), and the standardized path coefficient of SSR → JS is 0.046 (*p* = 0.204). Therefore, the direct effect of SSR on TW is significantly positive at the 0.01 level, while the direct effect of SSR on JS is not significant. The standardized path coefficient from SSR to IN is 0.596 (*p* < 0.01), the standardized path coefficient from IN to TW is 0.275 (p < 0.01), and the standardized path coefficient from IN to JS is 0.938 (*p* < 0.01). Therefore, IN shows a significant positive mediating effect in the relationships between SSR and TW as well as between SSR and JS. The standardized path coefficient from SSR to JB is −0.906 (*p* < 0.01), the standardized path coefficient from JB to TW is −0.15 (*p* < 0.01), and the standardized path coefficient from JB to JS is −0.027 (*p* < 0.01). Therefore, the mediating effect of JB between SSR and TW is significantly positive, but the mediating effect of JB between SSR and JS is not significant. Finally, JD_SSR denotes the interaction term between JD and SSR. The path coefficient of JD_SSR → IN is −0.18 (*p* < 0.01), and the path coefficient of JD_SSR → JB is 1.057 (*p* < 0.01). Therefore, JD negatively moderates the relationship between SSR and IN, and positively moderates the relationship between SSR and JB.

**Table 4:** The results of latent moderated structural equations model.

Path	Non-standardized coefficient	Standardized coefficient	Standard error	*Z*	*P*
SSR → TW	0.291	0.322	0.05	5.815	0.000***
IN → TW	0.313	0.275	0.063	4.983	0.000***
JB → TW	−0.176	−0.15	0.057	−3.064	0.002***
SSR → JS	0.029	0.046	0.023	1.271	0.204
IN →JS	0.753	0.938	0.075	10.072	0.000***
JB → JS	−0.023	−0.027	0.027	−0.842	0.400
SSR → IN	0.473	0.596	0.051	9.361	0.000***
JD_SSR → IN	−0.056	−0.18	0.018	−3.142	0.002***
SSR → JB	−0.698	−0.906	0.054	−12.818	0.000***
JD_SSR → JB	0.319	1.057	0.029	11.017	0.000***

***, **, and * represent significance levels of 1, 5, and 10%, respectively.

In summary, the empirical findings support all hypotheses except H1 and H4 which are not supported by the data, while H6 remains inconclusive and warrants further investigation.

### 4.4 The results of the mediating effect test

The estimation results of the structural equation model are susceptible to the sample distribution and sample size, so the mediating effects of IN and JB may need further verification. We examined the mediating effects of IN and JB between SSR and JS through a parallel mediation effect model using the bootstrap method with 1,000 repeated samplings. Then, we examined the mediating effects of IN and JB between SSR and TW through a parallel mediation model using the bootstrap method with 1,000 repeated samples. Running SPSS 27.0, we obtained the statistical results as shown in Table 5. The results indicated that the mediating effects of IN and JB between SSR and JS were 0.266 (*p* < 0.01) and 0.027 (*p* < 0.05) respectively, so the parallel mediating effect was significantly positive. Furthermore, the research findings also indicate that the mediating effects of IN and JB between SSR and TW are 0.107 (*p* < 0.01) and 0.044 (*p* < 0.05) respectively. Therefore, the parallel mediating effects of IN and JB between SSR and TW are significantly positive. Therefore, H6, H7, H11 and H12 are accepted.

**Table 5:** The results of the mediation effect test.

Path	Total effect	Direct effect	Indirect effect	Boot SE	*Z*	*P*	95%BootCI
SSR= > IN= > JS	0.442	0.149	0.266	0.03	8.919	0.000	0.209–0.327
SSR= > JB= > JS	0.442	0.149	0.027	0.011	2.576	0.010	0.007–0.048
SSR= > IN= > TW	0.467	0.316	0.107	0.025	4.256	0.000	0.061–0.161
SSR= > JB= > TW	0.467	0.316	0.044	0.017	2.586	0.010	0.015–0.084

### 4.5 The results of the moderating effect test

**Figure 2:**
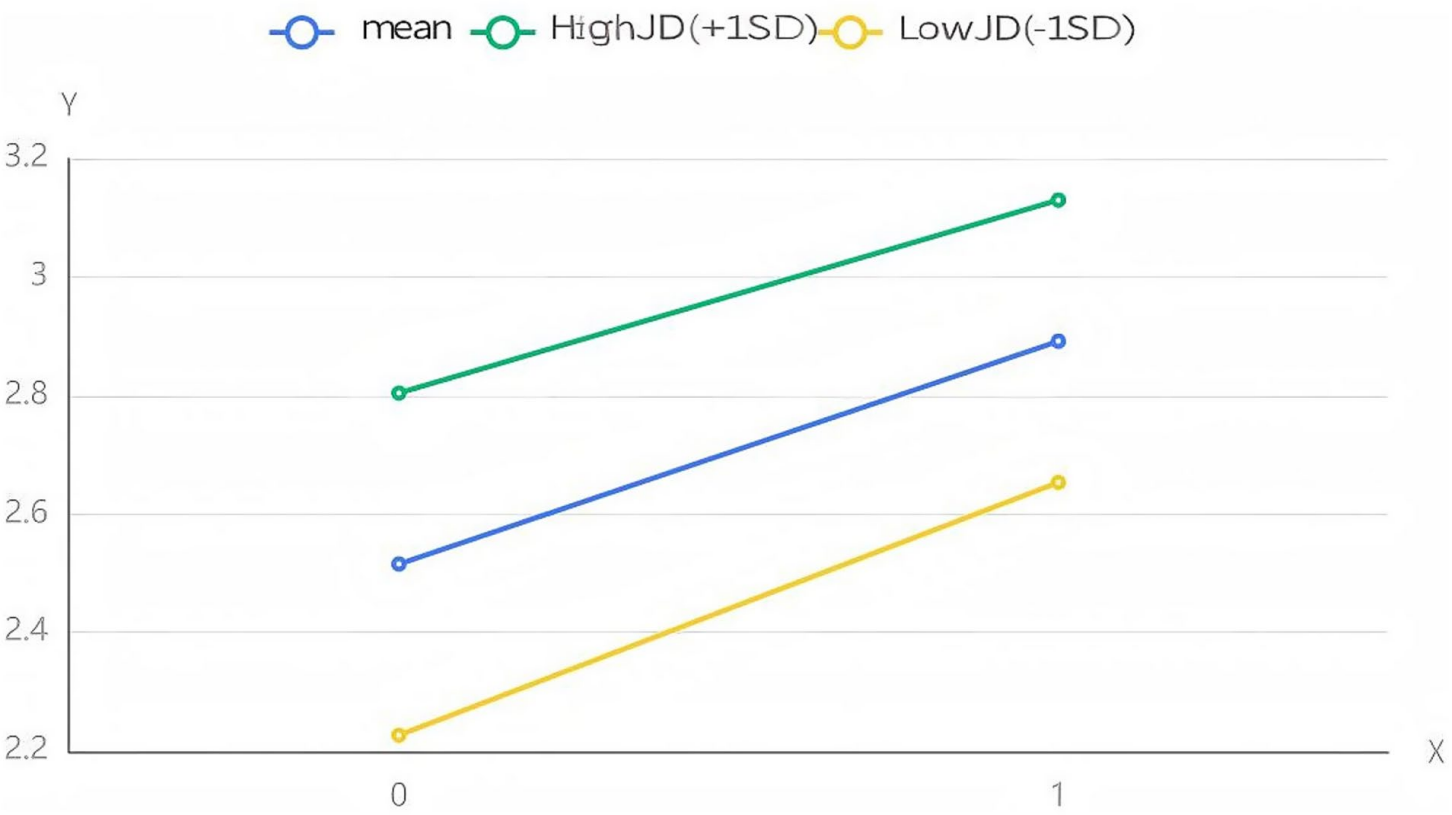
The moderating effect of JD between SSR and IN.

**Figure 3:**
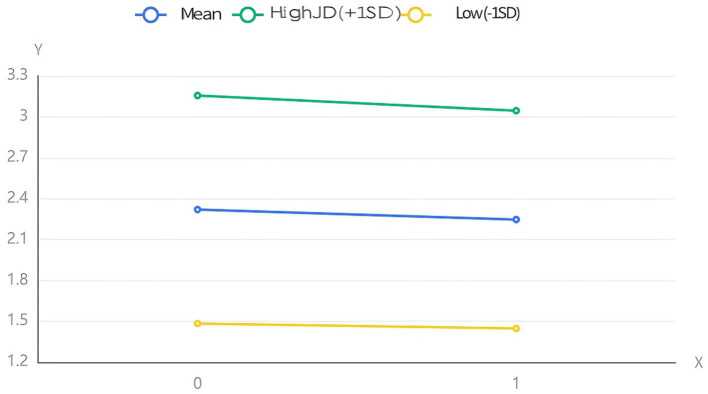
The moderating effect of JD between SSR and JB.

Running SPSS 27.0, we examined the moderating effect of JD on the relationship between SSR and IN under the condition of 1,000 repeated samplings. The result showed that the regression coefficient of the interaction term between SSR and JD was 0.059 (*p* > 0.10), indicating that the moderating effect of JD on the relationship between SSR and IN was not significant. We also examined the moderating effect of JD on the relationship between SSR and JB under the condition of 1,000 repeated samplings. The result showed that the regression coefficient of the interaction term between SSR and JD was 0.061 (*p* < 0.10), indicating that the moderating effect of JD on the relationship between SSR and JB was significant at the 0.10 level. Given this, H13 was accepted but H14 was rejected. To better present the above conclusion, we adopt a simple slope plot to reflect the moderating effect of JD (Figures 2, 3).

## 5 Discussion

### 5.1 Conclusion

To clarify the relationship between SSR and the wellbeing of employees in the workplace, the research team conducted a questionnaire survey of 429 employees and analyzed the sample data using latent moderated structural equation modeling (LMS). We have reached the following three conclusions:

(1) SSR has a positive direct impact on employees’ thriving at work but it does not have a significant direct impact on employees’ job satisfaction. Zhu et al. (2025) found a positive impact of SSR on employees’ subjective wellbeing when testing the predictive validity of the new scale. John and Ryan (2025) discovered the positive influence of SSR on workplace wellbeing through a pilot evaluation method (John and Ryan, 2025). Sabine et al. (2024) supported the correlation between SSR and employee wellbeing using tracking data (Sabine et al., 2024). To clarify the differentiated effects of SSR on different types of wellbeing, we distinguished between hedonic wellbeing and eudaimonic wellbeing, further supporting the positive role of SSR on thriving at work but not on job satisfaction. This result indicates that job burnout and intrinsic needs play a partial mediating role between SSR and thriving, and a full mediating effect between SSR and job satisfaction. According to the JD-R model, SSR can also increase cognitive resources and psychological resources by reducing cognitive load and physical fatigue, thereby promoting employees’ thriving at work (John and Ryan, 2025; De Beer et al., 2024). Therefore, intrinsic needs and job burnout are only partial mechanisms through which SSR affects the sense of thriving (Zhu et al., 2025; Porath et al., 2022). According to the definition of job satisfaction, employees form the final experience result by evaluating the positive and negative features of their work (Cammann et al., 1983; Judge et al., 1998). Based on JD-R model and SDT, the psychological consequences of negative features are mainly reflected in job burnout, while the psychological consequences of positive features are mainly manifested through the satisfaction of intrinsic needs (Bakker and Demerouti, 2024; Deci et al., 2017). Therefore, intrinsic needs and job burnout can basically fully mediate the impact of SSR on job satisfaction.

(2) Job burnout and intrinsic needs, respectively, mediate the relationships between SSR and job satisfaction and the relationship between SSR and thriving at work. Although Zhu et al. (2025), John and Ryan (2025), and Sabine et al. (2024) all analyzed the relationship between SSR and workplace wellbeing, these studies did not elaborate on the mechanism of SSR. Our conclusion further responds to the perspectives of the JD-R model and self-determination theory, finding that SSR mainly influences job satisfaction and thriving at work through two pathways: alleviating job burnout and fulfilling intrinsic needs. In emerging markets, manufacturing enterprises are advancing multiple transformation initiatives—such as digitalization, servitization, and ecologicalization—at an intense, catch-up pace (Urban and Plattfaut, 2025; Fan et al., 2014; Xu et al., 2022). While performing productive labor, workers are simultaneously required to exert a significant amount of emotional labor (Lee et al., 2024; Green, 2025; Townsend, 2008). In this situation, insufficient intrinsic motivation and elevated levels of work burnout among employees have emerged as primary barriers to enhancing subjective wellbeing (Wang et al., 2025; Yin et al., 2022; Ji et al., 2025; Çekmecelioglu et al., 2025). SSR has committed to full recovery practices, enhancing employees’ individual resilience, optimism and interpersonal relationships. In this sense, job burnout and intrinsic needs, respectively, play parallel mediating roles between SSR and job satisfaction as well as between SSR and thriving at work.

(3) Job demand positively moderates the relationship between SSR and job burnout but it does not significantly moderate the relationship between SSR and intrinsic need. Empirical research on the boundary conditions of sustainable self-regulation (SSR) remains scarce, with only (Sabine et al., 2024) examining the moderating role of leader-member exchange (LMX) in the relationship between SSR and wellbeing. Our findings indicate that job demands do not significantly moderate the association between SSR and intrinsic need satisfaction; however, they positively moderate the relationship between SSR and job burnout. This result aligns with the JD-R model, which posits that the effectiveness of SSR in reducing job burnout depends on supportive external work conditions (Bakker and Demerouti, 2024). In contexts of high job demands, the buffering effect of SSR on burnout is attenuated, suggesting that excessive workload may undermine the protective function of SSR. We apply the flow theory to explain the phenomenon that the moderating effect of job demands on the relationship between SSR and intrinsic needs is not significant. Although SDT regards work requirements as an external control which may weaken intrinsic needs (Deci et al., 2017), appropriate job demands can enhance the challenge inherent in work, elevate employees’ arousal levels, and potentially trigger a flow experience (Zito et al., 2016). The flow theory implies that the impact of job demands may be nonlinear and even be disrupted by other factors (Kasa et al., 2020; Vargic et al., 2023).

### 5.2 Management implications

Based on the research conclusions of the previous section, we have put forward three management suggestions for the organizational practices of SSR. First, enterprises should systematically introduce the four measures of SSR and comprehensively promote the implementation of SSR in a complete and best-practice-compliant manner. Although our findings indicate that SSR positively enhances workplace wellbeing, Zhu et al. (2025) argue that SSR can only realize its full potential when implemented as an integrated set of best practices within the organization. Fragmented or piecemeal SSR initiatives have limited impact on employees’ recovery experiences and may even foster skepticism among employees regarding the organization’s genuine commitment to promoting SSR. Second, when implementing SSR, organizations should foster employee autonomy, expand opportunities for social interaction among colleagues, and cultivate a relaxed work environment to reduce employees’ perceived stress. Following SSR implementation, employees are likely to experience an increase in non-work time. Grounded in the principles of satisfying intrinsic needs and alleviating job burnout, companies should encourage employees to use this additional time for social engagement, personal development, and self-directed activities, thereby facilitating recovery through a supportive and low-pressure organizational climate. Thirdly, when promoting SSR, enterprises should identify the differentiated needs of different work groups, especially paying attention to the urgency of employees in high-pressure positions. Under high-intensity workloads, the role of SSR in alleviating job burnout may be weakened. Enterprises should reasonably adjust the intensity of tasks during working hours and coordinate them with recovery activities during non-working hours, clearly demarcating the boundaries between working and rest periods to prevent work tasks from encroaching on non-working hours, thereby ensuring the restorative efficacy of SSR.

### 5.3 Contributions and limitations

This study contributes two incremental advances to SSR scholarship. First, at the empirical level, it distinguishes between distinct dimensions of workplace wellbeing and elucidates the underlying mechanisms and contextual boundaries through which SSR influences employee wellbeing, thereby strengthening the evidence base for evidence-based management of SSR. Second, from a theoretical standpoint, the study responds to the growing emphasis in positive organizational behavior on simultaneously addressing negative states and fostering positive motivations by proposing a dual-path model of SSR’s impact on workplace wellbeing, thus extending the theoretical framework of dual integration in positive organizational scholarship.

There are two main limitations in this study. First, the cross-sectional design limits temporal inference. While Sabine et al. (2024) employed a longitudinal tracking approach to reveal the long-term effects of SSR on employee emotions and behaviors, this study relies on cross-sectional data due to time and resource constraints. As a result, the findings may reflect situational characteristics at a specific point in time and are unable to capture dynamic developmental processes or changes over time. Second, causal inference is constrained. Although SSR, as an organizational-level best practice, can be implemented through quasi-experimental designs to enhance internal validity, this study employs a questionnaire-based survey method, which primarily detects associations among variables and offers weaker causal evidence compared to experimental or longitudinal approaches. In the subsequent research, we plan to adopt quasi-experimental and longitudinal data tracking methods to conduct empirical research, in order to make up for the above-mentioned limitations.

## Data Availability

The raw data supporting the conclusions of this article will be made available by the authors, without undue reservation.

## References

[ref1] Alwahhabi N. Dukhaykh S. Alonazi W. B. (2023). Thriving at work as a mediator of the relationship between transformational leadership and innovative work behavior. Sustainability 15:11540. doi: 10.3390/su151511540

[ref2] Bakker A. B. Demerouti E. (2017). Job demands-resources theory: taking stock and looking forward. J. Occup. Health Psychol. 22, 273–285. doi: 10.1037/ocp0000056, 27732008

[ref3] Bakker A. B. Demerouti E. (2024). Job demands-resources theory: frequently asked questions. J. Occup. Health Psychol. 29, 188–200. doi: 10.1037/ocp0000376, 38913705

[ref4] Bakker A. B. Demerouti E. Sanz-Vergel A. Rodriguez-Munoz A. (2023). Job demands-resources theory: new developments over the last decade. J. Work Organ. Psychol. 39, 157–167. doi: 10.5093/jwop2023a17

[ref5] Bernini C. Tampieri A. (2025). Disentangling job satisfaction: the roles of monetary and non-monetary factors across job types and income levels. Br. J. Ind. Relat. doi: 10.1111/bjir.70013

[ref6] Cammann C. Fichman M. Jenkins D Klesh J. (1983). The Michigan organizational assessment questionnaire. Assessing organizational change a guide to methods measure and practices. New York: John Wiley & Sons.

[ref7] Çekmecelioglu H. G. Balkas J. Altas S. S. Güler D. S. (2025). The effect of health professionals' perceptions of organizational impediments on emotional labor and job satisfaction. Front. Psychol. 16:1537830. doi: 10.3389/fpsyg.2025.1537830 40212315 PMC11983568

[ref8] Chen C. W. Zhang K. S. Chen C. M. Pao L. S. (2023). The impact between motivational potential characteristics of job and job satisfaction: a moderation model of personality traits in a high-tech industry. Int. J. Asian Bus. Inform. Manag. 14:325650. doi: 10.4018/IJABIM.325650

[ref9] Choubey A. P. Agrawal A. K. (2025). Theoretical foundation for integrating employee proficiency in job demands-resources theory. Int. J. Organ. Anal. doi: 10.1108/IJOA-03-2025-5348

[ref10] Criswell E. (2003). A challenge to humanistic psychology in the 21st century. J. Humanist. Psychol. 43, 42–52. doi: 10.1177/0022167803043003004

[ref11] De Beer L. T. Christensen M. Sorengaard T. A. Innstrand S. T. Schaufeli W. B. (2024). The psychometric properties of the burnout assessment tool in Norway: a thorough investigation into construct-relevant multidimensionality. Scand. J. Psychol. 65, 479–489. doi: 10.1111/sjop.12996, 38146078

[ref12] De Beer L. T. Schaufeli W. B. De Witte H. Hakanen J. J. Kaltiainen J. Glaser J. . (2024). Revisiting a global burnout score with the burnout assessment tool (BAT) across nine country samples. Eur. J. Psychol. Assess. doi: 10.1027/1015-5759/a000839

[ref13] Deci E. L. Olafsen A. H. Ryan R. M. (2017). Self-determination theory in work organizations: The state of a science. Annu. Rev. Organ. Psych. Organ. Behav. 4, 19–43. doi: 10.1146/annurev-orgpsych-032516-113108

[ref14] Deci E. L. Ryan R. M. (1985). Intrinsic motivation and self-determination in human behavior. New York, NY: Plenum Press.

[ref15] Demerouti E. Bakker A. B. Nachreiner F. Schaufeli W. B. (2001). The job demands-resources model of burnout. J. Appl. Psychol. 86, 499–512. doi: 10.1037/0021-9010.86.3.499, 11419809

[ref16] DeRobertis E. M. (2015). A neuroscientific renaissance of humanistic psychology. J. Humanist. Psychol. 55, 323–345. doi: 10.1177/0022167814536617

[ref17] Fan L. Zhu Y. M. Liu M. (2014). “An empirical research on influence of urbanization and industrialization on Servitization in Yangtze River Delta region” in Asia-Pacific Management and Engineering Conference (APME 2014), 592–598.

[ref18] Galanis P. Katsiroumpa A. Vraka I. Siskou O. Konstantakopoulou O. Katsoulas T. . (2025). The influence of job burnout on quiet quitting among nurses: the mediating effect of job satisfaction. Int. J. Nurs. Pract. 31:e70057. doi: 10.1111/ijn.70057, 40937525

[ref19] Ghazzawi R. Bender M. He J. Daouk-Öyry L. van der Heijden B. (2025). Examining the interplay between job crafting and job satisfaction: a cross-cultural investigation. Int. J. Cross-cult. Manag. 25, 507–529. doi: 10.1177/14705958251340537

[ref20] Green J. F. (2025). (Self-)seduction in the manufacturing of consent: exploring emotional exploitation in the service sector. Labour Indust. 35, 257–276. doi: 10.1080/10301763.2025.2549187

[ref21] Han X. Li Y. H. Li J. (2024). Having fun and thriving: the impact of fun human resource practices on employees' autonomous motivation and thriving at work. Hum. Resour. Manag. 63, 813–828. doi: 10.1002/hrm.22228

[ref22] Ji X. D. Wang F. Han Y. Song D. Chen Y. (2025). Emotion-focused human resource management and compassionate care behavior: integrating emotion regulation theory with public service motivation theory. J. Nurs. Manag. 2025:7902093. doi: 10.1155/jonm/7902093 41132753 PMC12543497

[ref23] Jiang Z. Jiang Y. Q. Nielsen I. (2019). Workplace thriving in China. Int. J. Manpow. 40, 979–993. doi: 10.1108/IJM-08-2018-0256

[ref24] John N. Ryan L. (2025). Strengthening leadership to support employee recovery: a pilot evaluation of the recovery friendly leader training program. Workplace Health Safety. doi: 10.1177/21650799251356304, 40767432

[ref25] Judge T. A. Locker E. A. Durham C. C. Kluger A. N. (1998). Dispositional effects on job and life satisfaction. J. Appl. Psychol. 83, 17–34. doi: 10.1037/0021-9010.83.1.17, 9494439

[ref26] Karadas G. Hassanie S. Altun Ö. Ozturk A. Sesen H. Vatankhah S. (2025). Meaningful work, psychological well-being, thriving at work and patient aggression: testing a moderated-mediation model. Cogent Bus Manag 12:2571432. doi: 10.1080/23311975.2025.2571432

[ref27] Kasa M. Hassan Z. Ng J. Busari A. H. Nor N. N. M. (2020). Role of flow between job demand and job resources among the hotel employees in Sarawak. Int. J. Bus. Soc. 21, 168–182. doi: 10.33736/ijbs.3245.2020

[ref28] Kelava A. Nagengast B. Brandt H. (2014). A nonlinear structural equation mixture modeling approach for non-normally distributed latent predictor variables. Struct. Equ. Model. 21, 468–481. doi: 10.1080/10705511.2014.915379

[ref29] Klein A. Moosbrugger H. (2000). Maximum likelihood estimation of latent interaction effects with the LMS method. Psychometrika 65, 457–474. doi: 10.1007/BF02296338

[ref30] Kleine A.-K. Rudolph C. W. Zacher H. (2019). Thriving at work: a meta-analysis. J. Organ. Behav. 40, 261–279. doi: 10.31234/osf.io/k5xhg

[ref31] Lee J. E. Bae S. M. (2024). Examining the mediating effect of job satisfaction on the relationship between job autonomy and life satisfaction in early adulthood: a five-year data analysis conducted through latent growth modeling. Curr. Psychol. 43, 8963–8971. doi: 10.1007/s12144-023-05044-8

[ref32] Lee H. L. Kim J. H. Kang T. S. Lee G. R. Lee H. Y. Kim H. W. . (2024). Disparities in workplace hazards and organizational protection resources by Enterprise size: a National Representative Study of south Korean manufacturing workers. Saf. Health Work 15, 284–291. doi: 10.1016/j.shaw.2024.06.001, 39309279 PMC11410467

[ref33] Li C. C. Niu Y. S. Xin Y. Hou X. H. (2024). Emergency department nurses' intrinsic motivation: a bridge between empowering leadership and thriving at work. Int. Emerg. Nurs. 77:101526. doi: 10.1016/j.ienj.2024.101526, 39418925

[ref34] Lin C. P. Xian J. L. Li B. X. Huang H. M. (2020). Transformational leadership and employees' thriving at work: the mediating roles of challenge-hindrance stressors. Front. Psychol. 11:1400. doi: 10.3389/fpsyg.2020.01400 32655458 PMC7325323

[ref35] Martela F. (2025). Well-being as having, loving, doing, and being: an integrative organizing framework for employee well-being. J. Organ. Behav. 46, 641–661. doi: 10.1002/job.2862

[ref36] Maslach C. (1986). “Stress, burnout, and workaholism” in Professionals in distress: issues, syndromes, and solutions in psychology. eds. Kilburg R. R. Nathan P. E. Thoreson R. W. (Washington, DC: American Psychological Association), 53–75.

[ref37] Maslach C. Jackson S. E. (1981). The Measurement of experienced burnout. J. Organ. Behav. 2, 99–113. doi: 10.1002/job.4030020205

[ref38] Meijman T. F. Mulder G. (1998). “Psychological aspects of workload” in Handbook of work and organizational psychology: work psychology. eds. Drenth P. J. D. Thierry H., vol. 2 (Hove: Psychology Press), 5–33.

[ref39] Mercer Marsh Benefits (2023). Employee Benefits 2023 Workplace Well-being Report. Available online at: https://www.mercer.com.cn/about/newsroom/health-on-demand-2023/ (Accessed October 15, 2025).

[ref40] Ni X. D. Zeng Z. N. Zhou J. Y. (2023). The effect of thriving at work on work-family conflict: the mediating role of workaholism. Front. Psychol. 14:113470. doi: 10.3389/fpsyg.2023.1136470, 38078268 PMC10702575

[ref41] Okros N. Virga D. (2025). From social support to thriving at work via psychological capital: the role of psychosocial safety climate in a weekly study. J. Manag. Psychol. 40, 52–66. doi: 10.1108/JMP-07-2023-0409

[ref42] Paterson T. A. Luthans F. Jeung W. (2014). Thriving at work: impact of psychological capital and supervisor support. J. Organ. Behav. 35, 434–450. doi: 10.1002/job.1907

[ref43] Petts R. J. Carlson D. L. Fan W. (2025). Work-place mismatch, work-family conflict, and psychological well-being among parents. Soc. Ment. Health. doi: 10.1177/21568693251344326

[ref44] Porath C. L. Gibson C. B. Spreitzer G. M. (2022). To thrive or not to thrive: pathways for sustaining thriving at work. Res. Organ. Behav. 42:100176. doi: 10.1016/j.riob.2022.100176

[ref45] Porath C. Spreitzer G. Gibson C. Garnett F. G. (2012). Thriving at work: toward its measurement, construct validation, and theoretical refinement. J. Organ. Behav. 33, 250–275. doi: 10.1002/job.756

[ref46] Qamar F. Afshan G. Rana S. A. (2024). Sustainable HRM and well-being: systematic review and future research agenda. Manag. Rev. Q. 74, 2289–2339. doi: 10.1007/s11301-023-00360-6

[ref47] Ryan R. M. Deci E. L. (2000). Self-determination theory and the facilitation of intrinsic motivation, social development, and the facilitation of intrinsic motivation, social development, and well-being. Am. Psychol. 55, 68–78. doi: 10.1037/0003-066X.55.1.68, 11392867

[ref48] Sabine S. Ronit K. Laura V. (2024). Leader support for recovery: a multi-level approach to employee psychological detachment from work. J. Occup. Organ. Psychol. 97, 1762–1788. doi: 10.1111/joop.12538

[ref49] Schaufeli W. B. Taris T. W. (2014). “A critical review of the job demands-resources model: Implications for improving work and health” in Bridging occupational, organizational and public health: a transdisciplinary approach. Eds. Bauer G. F. Hämmig O. (Dordrecht: Springer Science + Business Media), 43–68.

[ref50] Semmer N. Zapf D. Dunckel H. (1995). “Assessing stress at work: a framework and instrument” in Work and health-scientific basis of progress in the working environment. eds. Svane O. Johansen C. (Luxembourg: Office for Official Publications of the European Communities).

[ref51] Serafim A. Veloso C. M. Rivera-Navarro J. Sousa B. Valeri M. (2024). Toward a scale to assess the emotional intelligence and internal marketing of business employees in Portugal. J. Organ. Chang. Manag. 37, 1214–1229. doi: 10.1108/JOCM-06-2023-0229

[ref52] Song X. H. Xiang M. R. Liu Y. Yu C. H. (2020). Relationship between job satisfaction and burnout based on a structural equation model. J. Occup. Environ. Med. 62, e725–e731. doi: 10.1097/JOM.0000000000002040, 33021514

[ref53] Steed L. B. Swider B. W. Keem S. Liu J. T. (2021). Leaving work at work: a meta-analysis on employee recovery from work. J. Manag. 47, 867–897. doi: 10.1177/0149206319864153

[ref54] To W. M. Gao J. H. Leung E. Y. W. (2020). The effects of job insecurity on employees' financial well-being and work satisfaction among Chinese pink-collar workers. SAGE Open 10:2158244020982993. doi: 10.1177/2158244020982993

[ref55] Townsend K. (2008). Do production employees engage in emotional labour? J. Ind. Relat. 50, 175–180. doi: 10.1177/0022185607085701

[ref56] Urban I. Plattfaut R. (2025). The interplay of digital responsibility and digital transformation: empirical insights from a Nationwide digital transformation. Inf. Syst. Front. doi: 10.1007/s10796-025-10610-5

[ref57] van Zyl L. E. Shankland R. Klibert J. (2024). The study demands and resources scale: psychometric properties, longitudinal invariance, and criterion validity. Front. Educ. 9:1409099. doi: 10.3389/feduc.2024.1409099 PMC8239153 34211424

[ref58] Vargic I. V. Glavas D. Rijavec M. (2023). Job crafting as a determinant of employees' job satisfaction, work-related flow and well-being. Drustvena Istrazivanja 32, 321–346. doi: 10.5559/di.32.2.07

[ref59] Wang Y. Fu W. Q. Xu S. S. (2025). Special education teachers' emotional labor strategies and its relationship with job burnout in China: the moderating role of psychological capital. Work J. Prevent. Assess. Rehabil. 81, 2582–2592. doi: 10.1177/10519815241311181, 39973639

[ref60] Wang L. L. Zhang L. X. Ju B. (2023). Sustainable vitality and learning: the connotation, scale, and heterogeneity of dualistic psychological thriving at work. Sustainability 15:10804. doi: 10.3390/su151410804

[ref61] Wei S. H. Ding H. J. Sun H. H. (2025). Interplay between teachers' affective well-being and thriving at work: a cross-lagged study. J. Happiness Stud. 26, 1–23. doi: 10.1007/s10902-025-00863-x39664799

[ref62] Xu J. Han L. M. Yin W. (2022). Research on the ecologicalization efficiency of mariculture industry in China and its influencing factors. Mar. Policy 137:104935. doi: 10.1016/j.marpol.2021.104935

[ref63] Yin Y. Y. Sun S. Y. Song L. L. Jin C. C. Wang Y. (2022). Emotional labour strategies and job burnout: a meta-analysis of Chinese employees. Asian J. Soc. Psychol. 26, 219–237. doi: 10.1111/ajsp.12554

[ref64] Zhu Z. Kuykendall L. Baines J. I. Zhang B. (2025). Clarifying the construct of supervisor support for recovery and its impact on employee recovery experiences. J. Manag. doi: 10.1177/01492063241311228

[ref65] Ziegler R. Schlett C. (2013). Forms of job satisfaction: studies on the validity of the self-classification method and on differences in job valence, job situation, and dispositional affectivity. Z. Arbeits 57, 51–76. doi: 10.1026/0932-4089/a000107

[ref66] Zito M. Cortese C. G. Colombo L. (2016). Nurses' exhaustion: the role of flow at work between job demands and job resources. J. Nurs. Manag. 24, E12–E22. doi: 10.1111/jonm.12284, 25612156

